# Iohexol plasma clearance in children: validation of multiple formulas and two-point sampling times

**DOI:** 10.1007/s00467-016-3436-z

**Published:** 2016-07-01

**Authors:** Camilla Tøndel, Bjørn Bolann, Cathrin Lytomt Salvador, Damien Brackman, Anna Bjerre, Einar Svarstad, Atle Brun

**Affiliations:** 1 Department of Pediatrics, Haukeland University Hospital, N-5021 Bergen, Norway; 2 Department of Clinical Medicine, University of Bergen, Bergen, Norway; 3 Department of Clinical Science, University of Bergen, Bergen, Norway; 4 Laboratory for Clinical Biochemistry, Haukeland University Hospital, Bergen, Norway; 5 Department of Medical Biochemistry, Oslo University Hospital, Oslo, Norway; 6 Department of Pediatrics, Oslo University Hospital, Oslo, Norway; 7 Department of Medicine, Haukeland University Hospital, Bergen, Norway

**Keywords:** Glomerular filtration rate, Child, Chronic kidney disease, Renal function, Method

## Abstract

**Background:**

In children, estimated glomerular filtration rate (eGFR) methods are hampered by inaccuracy, hence there is an obvious need for safe, simplified, and accurate measured GFR (mGFR) methods. The aim of this study was to evaluate different formulas and determine the optimal sampling points for calculating mGFR based on iohexol clearance measurements on blood samples drawn at two time points (GFR2p).

**Methods:**

The GFR of 96 children with different stages of chronic kidney disease (CKD) (median age 9.2 years, range 3 months to 17.5 years) was determined using the iohexol plasma clearance, with blood sampling at seven time points within 5 h (GFR7p) as the reference method. Median GFR7p was 65.9 (range 6.3–153) mL/min/1.73 m^2^. The performance of seven different formulas with early and late normalization to body surface area (BSA) was validated against the reference.

**Results:**

The highest percentage (95.8 %) of GFR2p within 10 % of the reference was calculated using the formula of Jødal and Brøchner–Mortensen (JBM) from 2009, with sampling at 2 and 5 h. Normalization to BSA before correction of the distribution phase improved the performance of the original Brøchner–Mortensen method from 1972; P10 of 92.7 % compared to P10 of 82.3 % with late normalization, and a similar result was obtained with other formulas.

**Conclusions:**

GFR2p performed well across a wide spectrum of GFR levels with the JBM formula. Several other formulas tested performed well provided that early BSA normalization was performed. Blood sampling at 2 and 5 h is recommended for an optimal GFR2p assessment.

## Introduction

Measurement of glomerular filtration rate (GFR) by iohexol plasma clearance was introduced in the 1980s [1] and has increasingly been applied due to safety and good performance [2–6]. Plasma clearance, in comparison to renal clearance, eliminates the errors linked to inaccurate urine collection [1, 5, 7–10] and can be calculated as the ratio between the amount of the injected substance and the area under the plasma concentration curve. The slope–intercept technique (i.e., one-pool technique) needing a minimum of two blood samples, has been broadly used as it eliminates the need for many blood samples and extensive clinical examination. Chantler et al.’s fixed constant method [Clearance (Cl) = 0.80 × Cl_1_ (mL/min/1.73 m^2^)] [11] has been shown to be inaccurate [12, 13], leading to the development of a second-order polynomial of the form *a*Cl_1_ + bCl_1_^2^, by Brøchner–Mortensen in 1972 (BMadult) which has been widely used in its original form as well as in different subsequent modifications [14–17]. Single-point methods have also been developed, but these have generally been shown to perform more poorly than the slope–intercept technique [18–20]. Importantly, in the original Brøchner–Mortensen formula, normalization to 1.73 m^2^ body surface area (BSA) was undertaken after correction for the distribution phase, i.e., after the completion of the entire GFR calculation. When the formula was modified for children, normalization to BSA was done before correction for the distribution phase (BMchild) [15]. Of note, the British Nuclear Medicine Guidelines recommend early BSA normalization both in children and adults and also suggested using average coefficient values of BMadult and BMchild (BMcombined) [17]. Despite these recommendations, some pediatric nephrology centers have published several studies using the original BMadult without early normalization in children [21, 22]. In 2007, Fleming developed a new formula that includes early BSA normalization and a constant factor (Flem) [12]. Jødal and Brøchner–Mortensen further refined this new formula (JBM), where the constant factor was replaced by a BSA-dependent factor [13, 23]. Schwartz and co-workers have proposed several new formulas; a modification of the BMchild formula (SAM), and a minor change of the JBM formula by introducing a constant factor (NSM) [5, 6, 24]. The studies of Brøchner–Mortensen’s group were performed with ^51^Cr-EDTA, the group of Fleming used ^99m^Tc-DTPA, and the Schwartz group used iohexol. GFR measurements with all these three substances are comparable those of inulin clearance. However, iohexol is the only method without ionizing radiation, making it the preferred substance in many centers, especially for pediatric patients [10].

A major and as yet unsettled issue is the optimal sampling time points for the slope–intercept technique; to date, no consensus has been reached regarding a recommendation for a GFR measurements method in children that is both feasible and less time-consuming. Some centers have chosen shorter procedures with the latter blood sampling as early as 3 h after the injection of iohexol [21, 25]. However, current knowledge on optimal time points is limited [9] and needs further investigation.

The purposes of our study were to: (1) assess the accuracy of the different formulas for measuring GFR (mGFR) in blood samples drawn at two time points (GFR2p) by comparison with reference iohexol plasma clearance measurements, (2) find the optimal time points for blood sampling within a feasible timeframe (i.e., last blood sampling 5 h after injection), and (3) examine the effect on GFR determination of early and late BSA correction, i.e., the _before_ and _after-_ versions (Table 1).

**Table 1:** Methodology of glomerular filtration rate calculations

Calculations and methods^a^	Formula^b^
Calculation of reference GFR (GFR7p)	
Absolute GFR7p (mL/min) [5]	GFR = Cl = I/(expA/α + expB/β)
BSA normalized GFR7p (mL/min/1.73 m^2^)	Cl_BSA_ = Cl × 1.73/BSA
Calculations of 2-point GFR (GFR2p)	
Cl_1_	Cl_1_ = I/ expA/α
BSA normalized Cl_1_	Cl_1_,_BSA_ = Cl_1_ × 1.73/BSA
BMadult_after_ (original)^c^ [14]	Cl = 0.9908 × Cl_1_ – 0.001218 × Cl_1_^2^
	Cl_BSA_ = Cl × 1.73/BSA
BMadult_before_ (adapted)	Cl_BSA_ = 0.9908 × Cl_1,BSA_ − 0.001218 × Cl_1,BSA_^2^
BMchild_before_ (original)^d^ [15]	Cl_BSA_ = 1.01 × Cl_1_, _BSA_ − 0.0017 × Cl_1, BSA_^2^
BMchild_after_ (adapted)	Cl = 1.01 × Cl_1_ – 0.0017 × Cl_1_^2^
	Cl_BSA_ = Cl × 1.73/BSA
BMcomb_before_ (original)^e^ [17]	Cl_BSA_ = 1.0004 × Cl_1,BSA_ – 0.00146 × Cl_1, BSA_^2^
BMcomb_after_ (adapted)	Cl = 1.0004 × Cl_1_ − 0.00146 × Cl_1_^2^
	Cl_BSA_ = Cl × 1.73/BSA
Flem_before_ (original)^e^ [12]	Cl_BSA_ = Cl_1,BSA_ /[1 + 0.0017 × Cl_1,BSA_]
Flem_after_ (adapted)	Cl = Cl_1_ /[1 + 0.0017 × Cl_1_]
	Cl_BSA_ = Cl × 1.73/BSA
JBM_before_ (original)^e^ [23]	Cl_BSA_ = Cl_1,BSA_ /1 + f_BSA_ × Cl_1,BSA_
	f_BSA_ = 0.00185BSA^−0,3^
JBM_after_ (original)^e^ [23]	Cl = Cl_1_ /1 + f × Cl_1_
	f = 0.0032BSA^−1.3^
	Cl_BSA_ = Cl × 1.73/BSA
SAM_before_ (original)^d^ [6]	Cl_BSA_ = 1.0019 × Cl_1,BSA_ − 0.001258 × Cl_1, BSA_^2^
SAM_after_ (adapted)	Cl = 1.0019 × Cl_1_ − 0.001258 × Cl_1_^2^
	Cl_BSA_ = Cl × 1.73/BSA
NSM_before_ (original)^e^ [24]	Cl_BSA_ = Cl_1,BSA_ /[1 + 0.0012 × Cl_1,BSA_/100]
NSM_after_ (adapted)	Cl = Cl_1_ /[1 + 0.0012 × Cl_1_/100]
	Cl_BSA_ = Cl × 1.73/BSA

^a^GFR7p, Reference method for measured glomerular filtation rate (mGFR) using the iohexol plasma clearance formula, with blood sampling at 7 time points within 5 h; GRF2p, mGFR based on iohexol clearance measurements on blood samples drawn at 2 time points; BSA , body surface area in m^2^ where BSA = 0.024265 × W^0.5378^ × H^0.3964^ (with W the body weight in kg and H the height in cm); Cl_1_, clearance of iohexol based solely on the slow phase of the elimination curve, using the slope–intercept technique, but without correction for the distribution phase; _before_, early BSA normalization; _after_, late BSA normalization. For further explanation, see the references cited next to the formula and refer to the Introduction

^b^I is the dose of iohexol in milligrams, expA is the intercept of the slow curve (elimination phase, log concentration vs. time), with α its corresponding slope; expB, the intercept of the fast curve (distribution phase), with β its corresponding slope

^c^Formula developed in adults

^d^Formula developed in children

^e^Formula developed in children and adults

## Patients and methods

### Patients

A total of 96 children with chronic kidney disease (CKD) were recruited for this study (ClinicalTrials.gov Identifier NCT01092260), of whom 54 were treated at Haukeland University Hospital, Bergen, Norway and 42 were treated at Oslo University Hospital, Oslo, Norway. The median age of the children (55 males, 41 females) was 9.2 years (range 3 months to 17.5 years), the median weight was 28.2 (range 6.6–84.6) kg, and the median height was 133.9 (range 59–177) cm. Median reference GFR based on seven blood sample time points (GFR7p) was 65.9 (range 6.3–153) mL/min/1.73 m^2^. The patients were distributed evenly over the different GFR stages, with 28, 27, 23, and 18 patients in CKD stage 1, 2, 3, and 4–5, respectively. None of the children enrolled in the study had edema.

## Methods

Iohexol was administered as Omnipaque® 300 mg I/mL (GE Healthcare, Oslo, Norway; i.e., 647 mg iohexol/mL) given in a dose adapted to body weight as follows: <10 kg, 1 mL; 10–20 kg, 2 mL; 20–30 kg, 3 mL; 30–40 kg, 4 mL; ≥40 kg, 5 mL Omnipaque®. The syringe with iohexol was weighed before and after the injection to an accuracy of 0.01 g. The dose of iohexol (in milligrams) was calculated by first multiplying the difference in syringe weight by the concentration of iohexol (647 mg/mL) and then dividing the product by the density of iohexol at room temperature (1.345 g/mL). The iohexol bolus was followed by an injection of 15 mL physiologic saline.

Blood samples (0.5 mL) were drawn from a different intravenous access at 10, 30, 120, 180, 210, 240, and 300 min after the injection of iohexol. In 29 of the 96 patients, the second blood sample was drawn after 60 min instead of 30 min. Blood was also obtained before the infusion of iohexol to exclude interference of other metabolites with the iohexol assay. The blood was allowed to stand for 30–60 min before being centrifuged at 1000–1300 *g* for 10 min. Serum was stored at −20 °C until analysis at one center (Haukeland University Hospital); the samples collected at the other center were sent frozen on dry ice for iohexol analysis.

Serum concentrations of iohexol were determined by high performance liquid chromatography. The concentration of iohexol was calculated from the area under the largest iohexol peak as compared to an internal calibration curve prepared for each set of samples. Calibrators were made up from an iohexol stock solution, 180 mg I/mL (i.e., 388 mg iohexol/mL, Omnipaque®, GE Healthcare), which were diluted in pooled normal plasma to 100, 50, and 10 μg/mL, respectively. Small aliquots of the calibrators were stored frozen at −20 °C in vials for up to 1 year. The accuracy of the method was assessed by an external quality assurance program (Equalis, Uppsala, Sweden), and the precision, calculated as the total coefficient of variation over several months, was 4.1 % at 10 mg/L, 3.8 % at 25–290 mg/L, and 3.3 % at >290 mg/L. The iohexol analysis is accredited by the Norwegian Accreditation and complies with the requirements of NS-EN ISO I5189.

### Calculations and statistics

The 7-point GFR (GFR7p) was calculated according to Sapirstein, as described by Schwartz et al. [5, 26] (Table 1). GFR was normalized to 1.73 m^2^ BSA by the ratio 1.73/BSA, using the formula of Haycock et al. [27]. The 2-point GFR (GFR2p) was calculated with the slope–intercept technique and corrected for the distribution phase as described by Brøchner-Mortensen [14], and was normalized to 1.73 m^2^ BSA [27]. Due to the great variability of body size in the pediatric population and differences between children and adults, we tested the impact of BSA normalization before or after the mathematical correction for the distribution phase [i.e., normalization interposed in the calculation (early) or undertaken *after* the entire GFR calculation is completed (late)]. Table 1 shows the formulas used in the evaluation.

The performances of the different formulas for GFR2p were compared. Accuracy was calculated as the percentage of patients with values within ±5, 10, and 15 % (P5, P10 and P15, respectively) of the reference method (GFR7p). Estimates of bias as a measure of trueness, as well as assessment of limits of agreement and correlation as measures of dispersion, were systematically performed. As a general measure of accuracy, the 95 percentile of deviations from the reference method (95POD), was also determined, with 90 % confidence intervals (CI) calculated by bootstrapping to provide estimates of variability [28] (Tables 2–4).

McNemar’s test for paired categorical variables was used to compare P10 values. For the evaluation of optimal blood sampling times, pairs of 2 and 3, 2 and 4, 2 and 5, and 3 and 5 h were chosen. As the time points 2 and 5 h were considered to be the best sampling times (Table 2), these were chosen for the comparison of the different methods (Table 3). Subanalyses were performed for age groups (<6, <10, and ≥10 years), BSA groups (<0.5, <1.0, and <1.45 m^2^), and stage of CKD (<30, 30 to <60, 60 to <90, and ≥90 mL/min/1.73 m^2^) (Table 4). The software packages Excel, Analyse-it V2.26 (both Microsoft Corp., Redmond, WA), and SPSS Statistics version 22 (IBM Corp., Armonk, NY) were used for statistical analysis.

## Results

Table 2 shows the evaluation of optimal time and interval for GFR2p blood sampling using the formulas JBM, BMadult_before_, and Flem_before_. The best results were obtained when blood samples were drawn at 2 and 5 h with JBM; the P10 at these time points was 95.8 % compared to <90 % with the three alternative sampling time points pairs (*p* < 0.05)). The 95POD value was lowest (9.8) when the GFR2p was calculated with JBM using blood drawn at 2 and 5 h (Table 2).

**Table 2:** Effect of different blood sampling times and intervals

Formula^a^	Sampling schemes (h)	Mean bias (95 % CI) (mL/min/1.73 m^2^)	95 % Limits of agreement (mL/min/1.73 m^2^)	*R*	P5 (%)^b^	P10 (%)^b^	P15 (%)^b^	95 Percentile of deviations (90 % CI) (%)^c^
BMadult_before_	2 and 3	−0.05 (−1.10 to 1.00)	−10.2 to 10.1	0.9877	60.4	79.2	89.6	26.4 (16.5–64.9)
BMadult_before_	2 and 4	0.30 (−0.60 to 1.19)	−8.4 to 8.9	0.9911	67.7	87.5	95.8	14.12 (11.7–18.5)
BMadult_before_	2 and 5	−0.20 (−0.70 to 1.09)	−8.5 to 8.9	0.9910	78.1	92.7	97.9	12.4 (8.9–17.0)
BMadult_before_	3 and 5	1.07 (−0.16 to 2.31)	−10.9 to 13.0	0.9840	56.3	86.5	95.8	15.2 (11.7–18.7)
Flem_before_	2 and 3	−0.94 (−1.99 to 0.11)	−11.1 to 9.2	0.9877	59.4	82.3	89.6	25.8 (16.6–50.0)
Flem_before_	2 and 4	−0.60 (−1.50 to 0.30)	−9.3 to 8.1	0.9911	70.8	88.5	95.8	13.7 (10.8–22.8)
Flem_before_	2 and 5	−0.69 (−1.59 to 0.21)	−9.4 to 8.0	0.9911	77.1	92.7	97.9	10.6 (9.8–16.1)
Flem_before_	3 and 5	0.19 (−1.04 to 1.41)	−11.6 to12.0	0.9837	63.5	86.5	95.8	14.4 (11.7–17.0)
JBM	2 and 3	−1.82 (−2.87 to −0.76)	−12.0 to 8.4	0.9879	56.3	82.3	89.6	25.5 (16.7–64.7)
JBM	2 and 4	−1.46 (−2.38 to −0.55)	−10.3 to 7.4	0.9913	64.6	89.6	96.9	14.1 (11.0–20.4)
JBM	2 and 5	−1.69 (−2.57 to −0.82)	−10.1 to 6.8	0.9919	74.0	95.8	99.0	9.8 (7.6–11.2)
JBM	3 and 5	−0.72 (−1.92 to 0.48)	−12.3 to 10.9	0.9840	63.5	88.5	95.8	14.6 (11.7–17.1)

Evaluation of optimal time and interval for blood sampling was investigated using four different sampling schemes after iohexol injection: 2 and 3, 2 and 4, 2 and 5, and 3 and 5 h, respectively. *N* = 96.patients. By comparison with the reference method (GFR7p), mean bias, 95 % limits of agreement, and correlation coefficient (*R*) were calculated

^a^The GFR (in mL/min/1.73 m^2^) was calculated by 2-point GFR using the formulas JBM, BMadult_before_, and Flem_before_

^b^Accuracy was assessed as P5, P10, and P15, which is the percentage of patients within ±5, 10, and 15 % of the reference method, respectively

^c^The 95 percentile of deviations (95POD) [with 90 % confidence interval (CI)] shows the maximum deviation for 95 % of the results

**Fig. 1:**
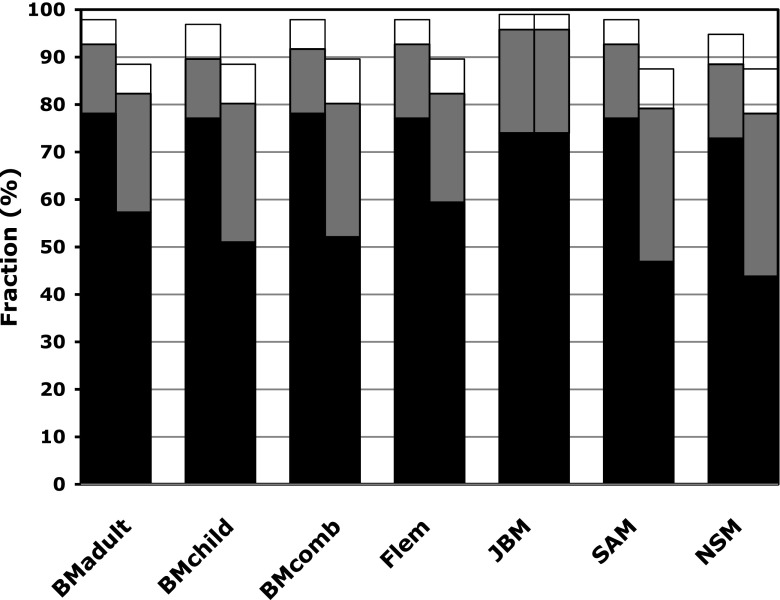
Accuracy of 7 different formulas for calculating measured GFR (mGFR) based on iohexol clearance measurements (see Table 1 for the formulas and the Introduction for more details). GFR (mL/min/1.73 m^2^) was calculated by 2-point GFR (GFR2p) using the different formulas as indicated and with blood sampling at 2 and 5 h after injection. *N* = 96 patients. *Color coding of graph*: *Black* Fraction of results within 5 % deviation of the reference method (GFR7p: GFR measured by the iohexol clearance method with blood sampling at 7 time points within 5 h), *gray* additional fraction within 10 % deviation of reference method, *white* additional fraction within 15 % deviation of reference method. *Left column of each column pair* Body surface area (BSA) normalization to 1.73 m^2^ performed “before” the mathematical correction for the distribution phase, *right column of each column pair* normalization performed “after” the entire GFR calculation was completed

The performance of the seven different formulas, used as presented in their original publication, is shown in Table 3. The performance of BMadult_before_ is also presented since this formula has been broadly recommended (Fleming et al. 2004). Figure 1 shows the accuracy of the various formulas and their *before* and *after* normalizations to BSA, as defined in Table 1.

All *before* formulas demonstrated high correlation factors (*r* ≥ 0.99), and relatively small mean biases of −2.02 to 2.15 mL/min/1.73 m^2^. Three formulas showed a high performance: the modified version of the classical BMadult (BMadult_before_), Flem_before_, and JBM. BM_before_ showed the smallest mean bias and the best 95 % limits of agreement as well as the highest P5, whereas JBM had the highest P10 score (Table 3). When the different formulas were evaluated by the 95POD, JBM showed the best value for accuracy. When BSA normalization was performed after the correction for the distribution phase, only JBM_after_ performed identically to JBM_before_ due to a separate and equivalent mathematical formula for this purpose in JBM. All of the other formulas performed substantially better with early compared to late BSA normalizations, i.e., *before* versions versus *after* versions (Table 3; Fig. 1). In general, GFR2p calculated with the *after* versions (GFR2p_after_) gave an overestimation of 2.88–5.03 mL/min/1.73 m^2^ (mean bias) compared to GFR7p, with relatively broad limits of agreement. The correlation factor was 0.979–0.985, and only 78.1–82.3 % of the results were within ±10 % of the reference method. All GFR2p_after_ had P5 and P15 of <60 and <90 %, respectively.

**Table 3:** Performance of different formulas

Formula^a^	Mean bias (95 % CI) (mL/min/1.73 m^2^)	95 % limits of agreement (mL/min/1.73 m^2^)	*R*	P5 (%)^b^	P10 (%)^b^	P15 (%)^b^	95 Percentile of deviations (90 % CI) (%)^c^
BMadult_after_	3.70 (2.38 to 5.02)	−9.1 to 16.5	0.9848	57.3	82.3	88.5	20.4 (16.3–24.5)
BMadult_before_	0.20 (−0.70 to 1.09)	−8.5 to 8.9	0.9910	78.1	92.7	97.9	12.4 (8.9–17.0)
BMchild_before_	−2.02 (−3.07 to −0.97)	−12.2 to 8.2	0.9900	77.1	89.6	96.9	11.9 (10.9–16.2)
BMcomb_before_	−0.92 (−1.86 to 0.02)	−10.0 to 8.2	0.9907	78.1	91.7	97.9	11.3 (9.2–16.5)
Flem_before_	−0.69 (−1.59 to 0.21)	−9.4 to 8.0	0.9911	77.1	92.7	97.9	10.6 (9.8–16.1)
JBM	−1.69 (−2.57 to −0.82)	−10.1 to 6.8	0.9919	74.0	95.8	99.0	9.8 (7.6–11.2)
SAM_before_	0.75 (−0.16 to 1.65)	−8.0 to 9.5	0.9910	77.1	92.7	97.9	13.2 (9.7–16.3)
NSM_before_	2.15 (1.17 to 3.12)	−7.3 to 11.6	0.9910	72.9	88.5	94.8	15.4 (12.5–16.8)

Glomerular filtration rate (GFR) (mL/min/1.73m2) was calculated by the 2-point GFR using the different formulas as indicated and with blood sampling at 2 and 5 h after injection. *N* = 96.patients. Mean bias (95% confidence interval), 95% limits of agreement, and correlation coefficient (*R*) were calculated by comparison with the reference method (GFR7p)

^a^The GFR (in mL/min/1.73 m^2^) was calculated by 2-point GFR using the formulas: JBM, BMadult_before_, and Flem_before_

^b^Accuracy, assessed as P5, P10, and P15, which is the percentage of patients within ±5, 10, and 15 % of the reference method, respectively

^c^The 95POD (with 90 % CI) shows the maximum deviation for 95 % of the results

**Fig. 2:**
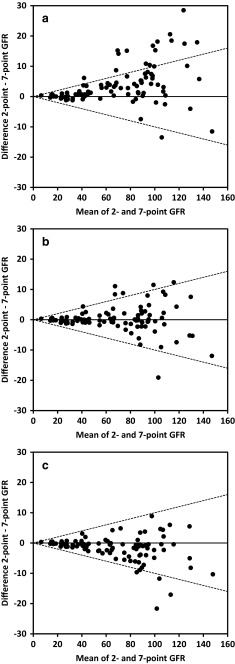
Bland–Altman plots between the glomerular filtration rate (GFR) (mL/min/1.73 m^2^) calculated according to reference method (GFR7p) and GFR calculated according to the BMadult_after_ formula (**a**), the BMadult_before_ formula (**b**), and the JBM formula (**c**). **a** GFR was calculated by GFR2p according to the 1972 formula of Brøchner–Mortensen (BMadult_after_) with sampling at 2 and 5 h after injection. BSA normalization to 1.73 m^2^ was performed after the entire GFR calculation was completed. *N* = 96 patients. *Y-axis* Difference between BMadult_after_ and the reference method (GFR7p), *X-axis* mean of the two methods. *Dotted lines* ±10 % difference. **b** GFR was calculated by GFR2p according to the 1972 formula of Brøchner–Mortensen (BMadult_before_) with sampling at 2 and 5 h after injection. BSA normalization to 1.73 m^2^ was performed before the mathematical correction for the distribution phase. *N* = 96 patients. *Y-axis* Difference between BMadult_before_ and the reference method (GFR7p), *X-axis* mean of the two methods. *Dotted lines* ±10 % difference. **c** GFR was calculated by GFR2p according to the 2009 formula of Jødal and Brøchner–Mortensen (JBM) with sampling at 2 and 5 h after injection. *N* = 96 patients. *Y-axis* Difference between JBM and the reference method (GFR7p), *X-axis* mean of the two methods. *Dotted lines* ±10 % difference

Difference plots of BMadult_after_ and BMadult_before_ are given in Fig. 2a, b; these demonstrate the importance of normalizing to 1.73 m^2^ before correction of the distribution phase in the widely used BMadult (*p* = 0.002 by McNemar’s test for P10) The difference plot of the formula that performs the best, i.e., JBM, is shown in Fig. 2c. When the results were classified according to different CKD stages, age, and BSA, the best accuracy in general was achieved by the JBM formula (Table 4).

**Table 4:** Subgroup analysis with glomerular filtration rate calculated using the formulas BMadult_before,_, BMchild, Flem_before_, and JBM

Patient groups	*N*	Method	Mean bias (95 % CI) (mL/min/1.73 m^2^)	95 % limits of agreement (mL/min/1.73 m^2^)	*R*	P5 (%)^a^	P10 (%)^a^	P15 (%)^a^	95 percentile of deviations (90 % CI) (%)^b^
All participants	96	BMadult_before_	0.20 (−0.70 to 1.09)	−8.5 to 8.9	0.9910	78.1	92.7	97.9	12.4 (8.9–17.0)
		BMchild_before_	−2.02 (−3.07 to −0.97)	−12.2 to 8.2	0.9900	77.1	89.6	96.9	11.9 (10.9–16.2)
		Flem_before_	−0.69 (−1.59 to 0.21)	−9.4 to 8.0	0.9911	77.1	92.7	97.9	10.6 (9.8–16.1)
		JBM	−1.69 (−2.57 to −0.82)	−10.1 to 6.8	0.9919	74.0	95.8	99.0	9.8 (7.6–11.2)
Age <6 years	33	BMadult_before_	1.13 (−0.34 to 2.61)	−7.0 to 9.3	0.9909	69.7	87.9	100	12.8 (9.7–13.4)
		BMchild_before_	−0.94 (−2.38 to 0.51)	−8.9 to 7.1	0.9914	75.8	90.9	100	11.1 (9.8–11.6)
		Flem_before_	−1.01 (−2.93 to 0.93)	−11.7 to 9.7	0.9870	69.7	87.9	97.0	13.1 (10.0–18.4)
		JBM	−2.00 (−3.62 to −0.39)	−10.9 to 6.9	0.9888	51.5	93.9	100	11.7 (8.0–14.0)
Age <10 years	52	BMadult_before_	0.88 (−0.48 to 2.23)	−8.7 to 10.4	0.9973	71.2	88.5	96.2	14.6 (11.3–17.9)
		BMchild_before_	−1.22 (−2.59 to 0.14)	−10.8 to 8.4	0.9879	76.9	90.4	96.2	12.9 (10.1–20.2)
		Flem_before_	−0.72 (−2.10 to 0.66)	−10.4 to 9.0	0.9892	71.2	90.4	96.2	12.7 (9.8–18.4)
		JBM	−1.99 (−3.35 to −0.63)	−11.6 to 7.6	0.9873	61.5	94.2	98.1	11.9 (7.8–19.2)
Age ≥10 years	44	BMadult_before_	−0.61 (−1.75 to 0.53)	−8.0 to 6.7	0.9948	86.4	97.7	100	8.8 (7.1–12.3)
		BMchild_before_	−2.96 (−4.62 to -1.31)	−13.6 to 7.7	0.9930	77.3	88.6	97.7	12.2 (10.1–16.2)
		Flem_before_	−0.67 (−1.82 to 0.49)	−8.1 to 6.8	0.9934	84.1	95.5	100	10.2 (7.3–11.6)
		JBM	−1.34 (−2.4 to −0.26)	−8.3 to 5.6	0.9957	88.6	97.7	100	9.63 (5.8–10.7)
BSA <0.5 m^2^	11	BMadult_before_	0.32 (−2.23 to 2.87)	−7.1 to 7.8	0.9938	63.6	90.9	100	NA
		BMchild_before_	0.98 (−2.49 to 4.45)	−9.1 to 11.1	0.9911	72.7	81.8	100	NA
		Flem_before_	−0.35 (−3.01 to 2.32)	−8.1 to 7.4	0.9939	72.7	90.9	100	NA
		JBM	−2.67 (−6.42 to 1.07)	−13.6 to 8.2	0.9921	45.5	90.9	100	NA
BSA <1.0 m^2^	47	BMadult_before_	1.31 (0.09–2.53)	−6.8 to 9.4	0.9915	72.3	89.4	97.9	13.0 (10.54–17.9)
		BMchild_before_	−0.66 (−1.82 to 0.50)	−8.4 to 7.1	0.9919	78.7	91.5	97.9	11.3 (9.6–15.2)
		Flem_before_	0.42 (−0.77 to 1.60)	−7.5 to 8.3	0.9914	74.5	91.5	97.9	11.3 (9.2–16.1)
		JBM	−1.69 (−2.90 to −0.48)	−9.7 to 6.4	0.9910	61.7	95.7	100	9.9 (7.6–14.0)
BSA <1.45 m^2^	77	BMadult_before_	0.59 (−0.44 to 1.62)	−8.3 to 9.5	0.9894	75.3	90.9	97.4	12.7 (9.3–17.01)
		BMchild_before_	−1.44 (−2.51 to −0.37)	−10.7 to 7.8	0.9895	77.9	90.9	97.4	11.7 (9.8 – 15.7)
		Flem_before_	−0.29 (−1.31 to 0.73)	−9.1 to 8.6	0.9893	76.6	92.2	97.4	11.0 (9.7–16.4)
		JBM	−1.70 (−2.73 to −0.68)	−10.6 to7.2	0.9895	70.1	94.8	98.7	10.7 (7.8–14.5)
GFR 30 mL/min/1.73 m^2^	18	BMadult_before_	−0.28 (−0.60 to 0.04)	−1.5 to 1.0	0.9960	94.4	100	100	NA
		BMchild_before_	−0.11 (−0.43–0.20)	−1.4 to 1.1	0.9960	94.4	100	100	NA
		Flem_before_	−3.00 (−0.63 to 0.03)	−1.6 to 0.99	0.9960	94.4	100	100	NA
		JBM	−0.36 (−0.69 to −0.03)	−1.7 to 1.0	0.9959	88.9	100	100	NA
GFR 30–59 mL/min/1.73 m^2^	23	BMadult_before_	0.21 (−0.40 to 0.83)	−2.6 to 3.0	0.9836	91.3	95.7	100	10.2 (4.7–11.3)
		BMchild_before_	0.01 (−0.60 to 0.62)	−2.8 to 2.8	0.9835	91.3	95.7	100	9.9 (4.6–10.9)
		Flem_before_	−0.15 (−0.76 to 0.46)	−2.9 to 2.6	0.9835	91.3	95.7	100	9.4 (4.5–10.4)
		JBM	−0.65 (−1.30 to 0.01)	−3.6 to 2.3	0.9817	87.0	100	100	7.6 (5.6–8.0)
GFR 60 to <90 mL/min/1.73 m^2^	27	BMadult_before_	0.90 (−0.62 to 2.43)	−6.6 to 8.4	0.9284	77.8	88.9	96.3	16.1 (9.9–17.9)
		BMchild_before_	−1.18 (−2.75 to 0.39)	−9.0 to 6.6	0.9284	74.1	92.6	96.3	13.5 (9.0–15.2)
		Flem_before_	−0.28 (−1.81 to 1.25)	−7.9 to 7.3	0.9284	77.8	88.9	96.3	14.3 (9.3–16.1)
		JBM	−1.83 (−3.11 to −0.55)	−8.2 to 4.5	0.9498	66.7	96.3	100	9.4 (7.1–10–7)
GFR ≥ 90 mL/min/1.73 m^2^	28	BMadult_before_	−0.20 (−3.00 to 2.61)	−14.4 to 14.0	0.8890	57.1	89.3	96.4	14.9 (10.3–17.0)
		BMchild_before_	−5.73 (−8.66 to −2.79)	−20.6 to 9.1	0.8824	57.1	75.0	92.9	18.4 (11.8–20.2)
		Flem_before_	−1.78 (−4.56 to 0.99)	−15.8 to 12.2	0.8914	53.6	89.3	96.4	14.8 (9.8–18.4)
		JBM	−3.28 (−5.98 to 0.57)	−16.9 to 10.4	0.8995	60.7	89.3	96.4	16.9 (9.7–19.2)

Glomerular filtration rate (GFR) (mL/min/1.73 m^2^) was calculated bythe 2-point GFR using the formulas: BMadult_before_, BMchild, Flem_before_, and JBM. Blood was sampled at 2 and 5 h after injection, and the results were subdivided according to age, BSA, and chronic kidney disease (CKD) stages as indicated. Mean bias (95 % CI), 95 % limits of agreement, and correlation coefficient (*R*) were calculated by comparison with the reference method (GFR7p)

NA, Not applicable due to insufficient data

^a^Accuracy was assessed as P5, P10, and P15, which is the percentage of patients within ± 5, 10 and 15 % of the reference method, respectively

^b^The 95POD (with 90 % CI) shows the maximum deviation for 95 % of the results

## Discussion

The results of our study show that the optimum time points for two-point blood sampling in children are 2 and 5 h after iohexol injection (Table 2). These time points are in accordance with the following mathematical and analytical considerations: a correct determination of the slope of the slow phase of the iohexol elimination curve is of crucial importance in GFR measurements based on the slope–intercept technique. When only two data points are used for a GFR calculation, the uncertainty in the assessment of this slope is dependent on both the separation distance between these two points and the analytical variation. With a short time-lapse between the two samplings, the difference between the data points is small, which implies that the analytical variation will contribute relatively more to the uncertainty of the slope than when the data points are well separated. A too close proximity in sampling times therefore introduces an unnecessary inaccuracy in the GFR determination, which was confirmed in our study by the high 95POD values of >25 for GFR2p based on sampling at 2 and 3 h . If sampling is undertaken too early, the elimination of iohexol has not yet reached the slow and linear phase, leading to an incorrect (too high) slope. Therefore, sampling which is too early is expected to result in an overestimation of the GFR, as has been shown in several studies [21, 25]. This is especially relevant at the lower GFR levels [5, 6, 24]. For clinical use, some centers will prefer to shorten the procedure and accept the cost of a lower accuracy, sampling at 2 and 3 h or at 2 and 4 h instead of 2 and 5 h. Other centers will choose to wait for 5 h after injection before collecting blood samples as long as this strategy results in a mGFR of higher quality.

Another important finding of our study is the dependency of the formulas on the timing of BSA normalization (Fig. 1; Table 3,), as recently discussed by Pottel et al. and Blake et al. [29–31]. A significant difference and substantially lower performance was found when BSA normalization was done after the correction for the distribution phase; for example, the accuracy of BMadult_after_ was clearly inferior to that of BMadult_before_ (*p* value of 0.002 for P10). The only exception was the JBM since it provides two mathematically equivalent formulas, one for normalization before and the other for normalization after, giving identical results [23]. The publication in 1972 by Brøchner–Mortensen of the correction for the distribution phase of the one-pool slope–intercept technique using a second-order polynomial [14] was a break-through in terms of simplicity and accuracy for GFR measurements. This method has been broadly used in its original form as well as in different modifications in both children and adults [9, 17, 20]. As early as in 1974 Brøchner–Mortensen et al. showed the importance of the different body sizes in children, and the pediatric formula was only meant to be used with early (before) normalization [15]. The study was based on a relatively small cohort of 30 children which may explain why some pediatric nephrologists have chosen to use the original method developed for adult patients (BMadult_after_), which was based on a considerably higher number of patients. It is of great importance that researchers are aware that the relatively low performance of the slope–intercept technique in some pediatric studies using BMadult_after_ is largely due to the use of BSA normalization after the entire GFR calculation was completed instead of being interposed in the calculation [21, 22]. In our view, this fact has not always been properly acknowledged and may be a source of erroneous conclusions in some previous studies.

All *before* formulas performed relatively well, with a P10 ranging from 88.5 to 95.8 % (Table 3). The JBM formula showed the best values for accuracy in this cohort, based on the highest P10 (95.8 %) and the lowest 95POD (9.8 %, 90 % CI 7.6–11.2). Based on the 95POD values with confidence intervals, the JBM formula performed significantly better than the recently published NSM_before_ formula (15.4 %, 90 % CI 12.5–16.8) (Table 3). The NSM_before_ formula [24] was meant to be an improvement of JBM. These two formulas are relatively similar, but the innovative BSA-dependent correction factor for the distribution phase in the JBM formula has been replaced by a constant in the NSM formula, which probably explains the latter’s lower performance. The BSA dependency of the correction factor was recently confirmed in a study of 142 children and adults using ^99m^Tc-DTPA–GFR [32]. JBM was also significantly better than the BMadult_after_ (Table 3), as well as the other *after* formulas (not shown). The other *before* formulas shown in Table 3 were not statistically different due to partly overlapping confidence intervals, but all showed higher 95POD values than JBM.

In the subgroup analysis according to age, BSA, and GFR (Table 4), the JBM formula showed the best accuracy in all groups except for age of <6 years and GFR of ≥90 mL/min/1.73 m^2^. For the age group of <6 years, the BMchild_before_ formula had the highest P5 and lowest 95POD, but the JBM formula showed the highest P10. Our findings suggest that these two formulas perform at a similar level. In the smaller children with a BSA of <1 m^2^, JBM had the best results, with the highest P10 and a lower 95POD compared to BMchild_before_, BMadult_before_, and Flem_before_, likely due to the BSA-dependent correction factor used in the JBM formula and not in any of the other formulas validated in this study.

A limitation of our study is the lack of the true gold standard marker inulin. However, inulin clearance is cumbersome and difficult to perform in children due to continuous intravenous infusion and timed urine collections, the latter also with a high risk of error, especially in children with urologic disorder, which is a common cause of CKD in the pediatric population [9]. Multipoint plasma clearance for GFR measurement is seen as a high-quality procedure and the “true” GFR with the last time point within a normal working day [10, 17, 33]. The last time point of iohexol measurement at 5 h may limit the value of the study in patients with a very low GFR, as true GFR may differ from the reference GFR. The number of patients in our study was limited to 96 children, and the subgroup analysis was therefore hampered by this low number of patients. However, the validity of our study is strengthened by comparisons of a high number of blood samples at different time points.

## Conclusion

The determination of GFR based on two-point iohexol plasma clearance performed well in children at all ages across a wide spectrum of GFR levels. The formula of Jødal and Brøchner–Mortensen from 2009 showed the highest percentage of GFR2p within 10 % of the reference GFR. Based on our findings, the optimum time points for blood sampling in children are 2 and 5 h after iohexol injection. The correct use of BSA normalization is essential for optimal GFR determination in children.
